# The Internet as Cognitive Enhancement

**DOI:** 10.1007/s11948-020-00210-8

**Published:** 2020-04-06

**Authors:** Cristina Voinea, Constantin Vică, Emilian Mihailov, Julian Savulescu

**Affiliations:** 1 Department of Philosophy and Social Sciences, Bucharest University of Economic Studies, Bucharest, Romania; 2 Department of Philosophy, University of Bucharest, Bucharest, Romania; 3 Oxford Uehiro Centre for Practical Ethics, University of Oxford, Oxford, UK; 4 Wellcome Centre for Ethics and Humanities, University of Oxford, Oxford, UK; 5 Murdoch Children’s Research Institute, Melbourne, Australia; 6 Melbourne Law School, Melbourne University, Melbourne, Australia

**Keywords:** Internet, Cognitive enhancement, Information overload, Misinformation, Persuasive design

## Abstract

The Internet has been identified in human enhancement scholarship as a powerful cognitive enhancement technology. It offers instant access to almost any type of information, along with the ability to share that information with others. The aim of this paper is to critically assess the enhancement potential of the Internet. We argue that unconditional access to information does not lead to cognitive enhancement. The Internet is not a simple, uniform technology, either in its composition, or in its use. We will look into why the Internet as an informational resource currently fails to enhance cognition. We analyze some of the phenomena that emerge from vast, continual fluxes of information–information overload, misinformation and persuasive design—and show how they could negatively impact users’ cognition. Methods for mitigating these negative impacts are then advanced: individual empowerment, better collaborative systems for sorting and categorizing information, and the use of artificial intelligence assistants that could guide users through the informational space of today’s Internet.

## Introduction

The Internet is a busy place. As of June 2018, almost half of the world’s population has access to the Internet^1^ (Internet Live Stats 2020). Each second, approximately 67 gigabytes of data flow through the network (Internet Live Stats 2020), with the average American user spending roughly 6 h online per day (Internet Live Stats 2020). Because of its decentralized and scalable infrastructure, the Internet democratized access to information, becoming one of the most successful communications and informational spaces to date. These characteristics contributed to the belief that we are witnessing a new technological Enlightenment that will radically transform the world for the better.^2^

The hope was that a global network will, one way or another, end up bettering the entirety of human existence, including cognition. As such, some have categorised the Internet amongst the most powerful cognitive enhancements technologies. For example, Bostrom and Sandberg claim that the “World Wide Web and e-mail are among the most powerful kinds of cognitive enhancement software developed to date” (Bostrom and Sandberg 2009, 311). The first reason for believing in the enhancement potential of the Internet is the fact that such external software and hardware offer humans “cognitive abilities that far outstrip those of biological brains” (2009, 312). The second reason is that the Internet’s wide diffusion and availability allows individuals from all over the world to collaborate and coordinate in an unprecedented manner for “the construction of shared knowledge and solutions” (2009, 322). Individual users gain access to information which they internalize, thus fortifying their expertise; and this knowledge can be further refined or reinforced when confronted with input from other users on the network: “Systems for online collaboration can incorporate efficient error correction enabling incremental improvement of product quality over time” (Bostrom and Sandberg 2009, 322). The assumption is that collaboration is helpful in sorting knowledge and in building more reliable systems of knowledge. If it weren’t for the informational dimension, the Internet would not be any different from other social institutions that help people coordinate and cooperate in order to share their knowledge and expertise—essentially, “conventional” means of cognitive enhancement (Bostrom and Sandberg 2009, 321). Thus, the Internet is considered the most powerful cognitive enhancement technology developed so far precisely *because* it complements the informational dimension with the collaborative one. It provides the possibility of instantly accessing almost any type of information, along with the possibility of instantly sharing it and connecting with others. By contrast, a social institution allows people to share what they *already* know. In other words, the Internet functions as an instantly accessible encyclopedia that opens the possibility for people bettering themselves, before engaging in social exchanges of information.

This paper takes a critical look at the claim that instant access to information offered through the Internet leads to cognitive enhancement. The Internet is not a simple, uniform technology, either in its composition, or in its use. There are many other characteristics of the online realm, besides information transmission and storage, which have serious implications for its enhancement potential. The quantity, structure and design of online informational resources matter for the cognitive enhancement potential of the Internet. Human beings can only process so much information at a given time. In rich informational environments, individuals take mental shortcuts in order to cope with complexity. This means that more information is not necessarily better, and might even be detrimental to users’ cognitive capacities. Cooperation could compensate for the shortcomings of individuals’ cognitive capacities, but we cannot simply assume that mere connection and interaction with other minds through the Internet will systematically enhance human cognition.

We analyze why the Internet as an informational resource fails to enhance cognition. Some of the phenomena that emerge from vast, continual fluxes of information are then analyzed—information overload, misinformation and persuasive design—for the purpose of showing how they could impact users’ cognition. We then propose methods through which these failings can be attenuated: individual empowerment, better collaborative systems for sorting and categorizing information, and the use of artificial intelligence assistants that could guide users through the informational space of today’s Internet. This critical assessment of the Internet as an informational resource can help us see where the Internet is failing us and where we can act in order to improve it and bring it closer to its cognitive enhancement potential.

## The Internet as a Cognitive Enhancement Technology

Cognition is hereby understood as the process of organizing and managing information and it includes acquiring (perception), selecting (attention), representing (understanding) and retaining (memory) information and using it to guide behavior (reasoning and coordination of motor outputs) (Bostrom and Sandberg 2009, 312). Cognitive enhancement thus is the increase or augmentation of the cognitive capacities of human beings. It refers, more precisely, to the creation of new cognitive capacities, or to the improvement of existing capacities within the normal range, or to raising the upper bound of the normal distribution (Buchanan 2011a, 146). The claim that the Internet is a tool for cognitive enhancement is usually situated within functional-augmentative approaches to enhancement (Earp et al. 2014). Within this framework, something would qualify as an enhancement “insofar as it improves some capacity or function (such as cognition, vision, hearing, alertness) by increasing the ability of the function to do what it normally does” (Earp et al. 2014).

When the Internet is understood as a cognitive enhancement, this is taken to mean that it helps individuals augment their existing cognitive capacities, like those of acquiring, processing and organizing information because it offers instant access to vast and varied amounts of information. Information is oftentimes unproblematically equated with knowledge. In Persson and Savulescu’s view, knowledge and its growth are enhancements because they “provide [individuals] with means of improving their standard of living” (Persson and Savulescu 2008, 163) by allowing them to pursue their ends in better and more efficient ways. Furthermore, they claim that “connection of minds and information through the Internet seem the most realistic means of substantial cognitive enhancement” (Persson and Savulescu 2008, 167). The feature that makes the Internet a distinctive technology, one with an enormous potential to enhance human cognition, is the connection between access to information and widespread social cooperation. Collaborative sharing and structuring of information can be cognitively advantageous, but only when information is already available to access and process. As such, access to information through the Internet is a prerequisite for its cognitive enhancement potential, which can then later be reinforced through collaborative processes of structuring and sharing information.

Similarly, Buchanan argues that computers and the Internet are some of the best technologies for cognitive enhancement because they open the possibility for instantly accessing information, anytime and anywhere (Buchanan 2011b, 9). He situates these two technologies within a long tradition of “historical non-biomedical cognitive enhancements”: literacy, (social) institutions and numeracy. All these interventions increased individuals’ cognitive capacities, expanding the landscape of intellectual tasks humans could perform (Buchanan 2011b, 24). In Buchanan’s view, too, the collaborative dimension of the Internet is important, but what distinguishes the Internet from literacy or social institutions is the fact that it opens the possibility for users to access whatever information they need, at any time they need it, which can increase knowledge production that can be later shared on the network.

The basic assumption questioned here is that more information is better in general because it leads to knowledge. Usually, information is taken to be good just by virtue of being information (Himma 2007, 259). As more information is available on the Internet, individuals will develop new forms of learning, which will increase the body of knowledge; knowledge production is enhanced by the collaborative dimension of the Internet; thus, more information is supposed to lead to more knowledge, which is further refined through collaboration.

This is not the only way of conceptualizing the enhancement potential of the Internet, as Heerminsk clearly shows (Heerminsk 2016; Smart et al. 2017). For example, the Internet affects users’ memory by constituting itself as a sort of external memory device (Sparrow et al. 2011). “The Google effect” refers to the fact that users store less information in their biological memory because they know that information is available on the Internet. Some argue that the Google effect is a beneficial adaptive mechanism that results in freeing up internal cognitive resources, a process also called ‘cognitive offloading’ (Ward and Wegner 2017; Risko and Gilbert 2016; Storm et al. 2017). However, others point out that the Internet encourages shallow information processing and reduced contemplation (Carr 2011). But there seems to be no sufficiently strong evidence to support any of these claims (Heersmink 2016).

Our analysis will not focus on storage of information, but on whether the Internet helps users develop better skills at finding, comparing and internalizing information, leading to knowledge formation. In the following sections, we analyze phenomena like information overload, misinformation and persuasive design in order to show how cognitive capacities could be put under great stress by continual fluxes of information. For this purpose, we start by assessing the effects of overloading the mind with information. We then look at the spread of misinformation and the confusion it generates in users who are unable to discern between reliable and unreliable information. This is followed by an analysis of the ways in which human attention, necessary for processing information, is exploited through the use of persuasive design. As it will be shown, the problems of information overload, misinformation and persuasive design force us to reconsider how we conceptualize the enhancement potential of the Internet, and consequently what is needed for genuine Internet-based cognitive enhancement.

## Is More Information Always Better? Overloading the Mind

The Internet has grown to be the biggest and most easily accessible source of information. Not only does it allow access to information, but it enables the creation of information at an unprecedented rate due to ever-increasing computing and processing capacities and the opportunities it creates for both human and non-human expression.

Although it is difficult to quantify all the information that is stored or sent across the Internet, researchers have tried to make some estimates. For example, as of November 2018, there are 5.28 billion Web-pages on the indexed Web (The Size of the World Wide Web 2018), excluding the Dark or Deep Web. According to Cisco’s Visual Networking Index, by 2019 the global traffic on the Internet will reach 2 zettabytes per year (Cisco Visual Networking Index: Forecast and Trends, 2017–2022). To get a sense of the quantity of information running through the Internet annually, a zettabyte is equivalent to 36,000 years’ worth of HD quality video. It is difficult to wrap our minds around these numbers, but the concept of information overload gains new significance due to the vast quantity of information, continually bombarding individuals through news, notifications, alarms and other visual or audio cues coming from smart devices.

The problem of information overload has received considerable attention from researchers concerned with the negative consequences of exposing individuals to too much information.^3^ This phenomenon is about individuals’ diminished ability to understand all the information within rich informational environments and, consequently, to cope with them. Humans are averse to uncertainty (Shannon 1948); we need information to decide and choose among several possibilities of action. This is both the strength and the Achilles’ heel of humans as information seekers: we ground our knowledge in information which makes us easily adaptive to an environment. The more the communication and information transmission channels grow, the more energy is spent to reduce uncertainty, thus overloading cognition.

Just like computers, human brains process information.^4^ Still, like any other information-processing systems, the human brain, although extremely complex, is limited in its processing capacity. Research in cognitive neurosciences highlights a number of bottlenecks impinging upon the flow of information from sensation to action (Anderson 2009, 63). These bottlenecks are human limits in perceiving all the relevant information in the environment, in holding it in mind and in acting upon the visual world (Marois and Ivanoff 2005). For example, human short-term memory is limited, the average adult being able to store approximately seven meaningful units (plus or minus two) at a time (Miller 1956). But it is not just short-term memory; *all* our cognitive subsystems have some upper limit in information processing (Marois and Ivanoff 2005), meaning that human brains have a limited processing capacity or bandwidth.

What happens when limited cognitive processing capacities are bombarded with information? Every new piece of information will actually contribute to a decrease in decision accuracy when a certain threshold is reached (Eppler 2015, 220). This is consistent with Gigerenzer and Brighton’s findings of “less-is-more” effects, more information actually resulting in a decrease in accuracy. This does not mean that the less information, the better the decision accuracy, but that there is “a point at which more information or computation becomes detrimental, independent of costs” (Gigerenzer and Brighton 2009, 111). When cognitive inputs become too complex, in terms of a subject receiving too much information, they will tend to take shortcuts so as to relieve the burden of complexity thus short-circuiting rational thought processes. More specifically, subjects will filter out information (typically choose the information that suits their worldview), will single out the puzzling information or will focus on the information that was received first (Eppler 2015, 220). In other words, information overload impairs decision-making in at least two ways: firstly, it makes it harder for individuals to locate the information due to sheer volume; secondly, it makes it difficult for individuals to locate the critically relevant information for a particular task (Herbig and Kramer 1994, 45). As such, in rich informational environments, information is hard to internalize. Thus, more information is not necessarily a guarantee that the subjects will gain a more comprehensive or clear understanding of a particular topic or that they will make decisions more efficiently.

The Internet contributes to the wide diffusion of the phenomenon of information overload. It made possible an explosion of information production which is unprecedented in human history. Given that more than half of the world population is now online, more information is produced, uploaded and processed daily than ever before in human history. This means that today’s Internet users deal, on a day-to-day basis, with much more information than their predecessors. Actually, “a weekly edition of the *New York Times* contains more information than the average person was likely to come across in a lifetime in seventeenth-century England” (Bawden and Robinson 2009, 5).

Besides the growth in channels for the production and transmission of information, the structure of information available online also has a large part to play in the pervasiveness of information overload.^5^ In traditional libraries, information is organized within subject areas and these subject areas, in their turn, are organized into a hierarchy by professionals trained to index and organize information. Librarians painstakingly describe and classify every new piece of information, so that it can coherently be integrated into a system which can be easily searched and accessed. The structure of information in a library is the result of expert knowledge. On the Internet anyone can upload, label or link any new piece of information to any other source of information already online. While this contributed to the democratization of expression and information access, it has also created informational spaces which are difficult to manage. Almost three and a half billion people access the Internet through social media platforms (Global Digital Report 2019, We Are Social). All the major digital platforms (Google, Facebook, Twitter etc.) work as gatekeepers, meaning that they curate and structure online information. The work of structuring information is usually done by algorithms (Gillespie 2014). These algorithms are not optimized to prioritize truthful information or to make it more prominent and easily accessible. They are built for engagement, pushing information which provokes outrage or sells the most advertising (Williams 2018). Thus, the information is structured for advertising optimization purposes or for user engagement, but this structuring process is opaque to the public because the algorithms underlying them are protected intellectual property (see, for example, Introna and Nissenbaum 2000; Gillespie 2014). As a result, the more the network grows, the more information is produced, transmitted and accessed the more difficult it becomes to trace truthful or useful information that might lead to knowledge. In the next section we further explore how the algorithmic structuring of information contributes to the proliferation of misinformation online and how it impacts users’ cognitive capacities.

## Misinformation

The overabundance of information is not the only characteristic of online information that burdens human cognition. In addition, information on the Internet is malleable. Anyone can easily upload new information on the network, or can update, alter, rewrite or even delete it. All the information on the Internet is roughly presented in the same format, a website or an app, whether on a computer, smartphone or any other device. This creates a psychological “levelling effect” which puts all the information online on the same accessibility and credibility level (Metzger and Flanagin 2013). The impossibility of relying on traditional verification mechanism—for example, information intermediaries such as experts, public intellectuals, categorization systems and so on, makes online information homogeneous. When all information looks alike it is very hard to say what is reliable. This is one of the reasons why the problems of mis- and disinformation^6^ are aggravated online.

In 2013, the World Economic Forum identified misinformation in online media as one of the most threatening phenomena faced by society (World Economic Forum—Global Risks 2013). Misinformation has been around forever. In the digital realm this phenomenon gains new amplitude because of “the convergence of social media, algorithmic news curation, bots, artificial intelligence, and big data analysis” (Yochai et al. 2018, 351). It is easier, faster and cheaper for anyone to send individually tailored information meant to manipulate users by using the latest research in behavioral sciences, such as psychographic marketing techniques.

A recent longitudinal study investigating the spread of true and false news online showed that falsehoods or fake news spread faster, deeper and more broadly than the truth. Fake news items imitate real news and are meant to spread virally while changing individuals’ beliefs (Rini 2017). The conclusion of the longitudinal study is stark. Falsehoods were 70% more likely to be retweeted than the truth (Vosoughi et al. 2018, 4). The most plausible explanation for the different diffusion of truth and falsity on the Internet is, actually, a human bias: attention is captured by novelty, and fake news, misinformation or disinformation are novel. As Vosoughi, Roy, and Aral explain, “Novelty attracts human attention, contributes to productive decision-making, and encourages information sharing because novelty updates our understanding of the world” (2018, 4).

Human attention is selective. In rich informational environments, attention will be drawn by the salient features of that environment, either as a function of the intrinsic qualities of that feature (for example, novelty), or as a function of the subject’s dispositions and attitudes (Taylor and Fiske 1978, 253). As such, Vosoughi, Roy, and Aral hypothesize that misinformation is the salient information in SNSs (social networking services) because it usually contains more novel information than accurate news reporting. Consequently, fake news induces the false belief in individuals that they are learning something ‘new’ about the world they live in. This inclination towards novelty builds upon another social bias, the in-group bias. By sharing novelties, individuals feel part of a group that has access to ‘unique’ information, which is seen as a shortcut to social status. As such, fake news spreads faster on the Internet because it feeds on two human vulnerabilities: the orientation of attention towards novelty and the illusion that sharing novelty confers a certain type of social status, making individuals feel part of a group of people who are “in the know”.

The fact that most information curation services deliver personalized information to each individual user, tailored to suit their pre-existing beliefs and preferences, produces concerns connected to echo chambers. Echo chambers are closed systems of like-minded people that have the effect of amplifying and reinforcing users’ beliefs, while isolating them from opposing views. This happens because information curation services work by instrumentalizing users’ irrational biases, like confirmation bias or cognitive dissonance (Garrett 2009). These biases show that people generally tend to find more pleasure in informational resources that confirm their already existing beliefs. In the digital context this means that users will pay more attention to information that reinforces their preferred narratives, such that they will generally seek, receive and share it, while avoiding exposure to opposing viewpoints and worldviews (Quattrociocchi et al. 2016). Although political, social and ideological segregation are a worrying effect of echo chambers, studies have shown that people’s inclination to avoid information challenging their views is much weaker than their inclination to seek information that reinforces their views (Flaxman et al. 2016; Garrett 2009). This means that people are not as averse to other perspectives as initially considered. As such, while social media and search engines are generally associated with ideological and political segregation, counterintuitively, they also massively contribute to exposing users to different viewpoints and worldviews (Flaxman et al. 2016, 21). While the effects of echo chambers have been largely inflated, they do pose a serious challenge to the enhancement potential of the Internet. Because of this phenomenon, it is increasingly difficult for individual users to correct their views or to engage with others in rational and disinterested debate. This configuration affects the quality of public discourse and diminishes collaboration between individuals, which is helpful in sorting knowledge or in building more reliable systems of knowledge.

## Distraction by Design

Attention is one of the most important cognitive resources. It allows us to select certain features of the environment that we will tend to, especially in rich informational environments, where reliable and unreliable pieces of information are hard to tell apart. We have to pay attention in order to make sense of information. The Internet’s structural connection with information and the fact that users are distracted by continual fluxes of data led to the creation of persuasive design. It refers to the designing techniques that exploit users’ psychological biases in order to capture their attention (Williams 2018).

Persuasive design exploits triggers, emotions and automatic cognitive scripts to produce non-reflective and sometimes irrational states of mind. Because of the Internet advertisement business models, we can now measure more things about individual human beings than ever before. James Williams compared these methods to a “Cambrian explosion” of advertisement measurements that can infer things such as “people’s behaviors (e.g. page views), intentions (e.g. search queries), contexts (e.g. physical locations), interests (e.g. inferences from users’ browsing behavior), unique identifiers (e.g. device IDs or emails of logged-in users), and more” (2018, 31). All this data revealing patterns in users’ behavior, coupled with the decision-making biases that were studied by behavioral psychologists and economists—like fear of missing out (Przybylski et al. 2013), framing effects, anchoring effects, social comparison and so on (Williams 2018, 33), allows digital industries to have a profound and measurable impact on our attention.

For example, one of the most successful and widely used features of social media platforms, infinite scroll feeds (like Facebook’s Newsfeed) which allows users to view content without a finishing line in sight, is based on one such cognitive bias. People respond faster, more efficiently and compulsively when the rewards they received for doing a particular task are intermittent. Dopamine is released not only when a certain task is accomplished and the associated reward is received, but also when there is anticipation of the rewards: “In other words, once reward contingencies are learned, dopamine is less about reward than about its anticipation” (Sapolsky 2017). This release of dopamine is what keeps users glued to social media platforms by captivating their attention and, consequently, what cultivates in them the habit of compulsively using these services.

Most persuasive design applications and websites work on a simple principle: in order to induce a certain type of behavior in users one has to offer them *a motivation*, *an ability* and *a trigger* for that behavior, according to a model developed by Fogg (2009). For example, social media platforms must keep people as long as possible on their platforms and must attract as many users as possible. In order to do this, they use the human desire for social acceptance and the fear of social rejection as *a motivation*. The *ability* to remain on the platform is through designing the interface to make engagement as easy and intuitive as possible, for example, by decreasing the number of clicks needed to accomplish a particular task, by offering it for free or by routinizing it. Nudging people into uploading photos (for example, Facebook constantly sends messages to users without a profile picture to remind them to complete the set-up process), into taggin, following, or otherwise connecting with one another (most social media platforms automatically suggest users that people should tag, follow, or connect with) or into checking their notifications (by sending emails or by using visual and audio cues to alert the user to a new notification) are just some of the *triggers* used. These types of triggers are called “facilitators” (Fogg 2009, 6) because they make a certain behavior easy to do. In Fogg’s words: “Triggers can cause us to act on impulse. For example, when Facebook sends me an email notification that someone has tagged me in a photo, I can immediately click on a link in that email to view the image. This kind of trigger-behavior coupling has never before been so strong” (2009, 7).

Persuasive design creates different types of distractions. The first ones are functional, meaning we are directed away “from information and actions relevant to our immediate tasks and goals” (Williams 2018, 50). This is the most common type of distraction and it happens, for example, when we interrupt our work flow in order to check email or other notifications. The second type of distractions are existential, in the sense that they instill actions and habits that diverge from users’ identity and values—for example, when users prioritize “likes” and “loves” over more meaningful real-life relationships (Williams 2018, 56). The third type of distraction is epistemic in its nature; Williams (2018, 68) refers here to the “diminishment of underlying capacities that enable a person to define or pursue their goals: capacities essential for democracy such as reflection, memory, prediction, leisure, reasoning, and goal-setting.” Epistemic distractions arise every time we find it difficult to discern reliable from unreliable information. Such distractions appeared only recently and perhaps the most salient example is that of “fake news”. All these types of distractions undermine people’s autonomy, by instilling habits and desires that are not voluntarily chosen. In other words, persuasive design steers users’ attention towards irrational behaviors.

## Towards a Robust Internet-Based Cognitive Enhancement: Three Cumulative Solutions

When confronted with big fluxes of information, humans will tend to pay attention to the novelty of information and not to its quality. They will short-circuit rational thought processes to deal with informational complexity and will eventually take shortcuts that can influence their decision-making capacities. In what follows we propose some solutions that can mitigate the problems of information overload, misinformation and persuasive design. These solutions are not strictly technological. For example, technology could help us extend our attention; for if a system cannot process all the information, it makes sense to expand its processing capacity rather than minimize the information flow. Our claim is that while technological solutions are necessary, they are not sufficient. This is the reason why the following strategies are aimed both at the individual users and the communities they are part of.

In order to mitigate the detrimental effects of information overload, misinformation and persuasive design, users need to have some understanding of how the different technological systems *interact* in order to control them. This is why purely technological solutions to the above problems are not enough. They must be complemented by traditional forms of user empowerment—through information literacy—and the creation of institutions meant to harness the “wisdom of the crowds” to apply it for purposes of information categorization, ranking and assessment. The strategies advanced are complementary—they aim at the individual, collective and technological level, each contributing to strengthening different weaknesses in users’ cognitive capacities.

Traditional forms of enhancement inspire the first strategy. Empowering users for better information retrieval should be grounded in the augmentation of user capabilities so that they can gain a better understanding of the Internet as an informational resource. In the same vein as non-biological forms of enhancement, which proved successful in extending the cognitive capacities of individuals, information literacy improves the capacity to navigate the virtual realm. The American Library Association defines it as a “a set of abilities requiring individuals to recognize when information is needed and have the ability to locate, evaluate, and use effectively the needed information” (Welsh and Wright 2010, 1). Information literacy aims at creating skills, based on critical reflection on the nature of information itself, which could be useful both on and offline (Bawden and Robinson 2002). Information literacy can be integrated into school or university curricula and courses in any number of ways but it can also be taught through informal methods like online video games. For example, *Factitious* is an online game meant to help players understand the difference between fake news and real news, while *Allies and Aliens* is another example of an online game “designed to increase students’ ability to recognize bias, prejudice and hate propaganda on the Internet and in other media” (Allies and Aliens website). The aim of information literacy is not to help individuals become information repositories or to burden them with even more information, but to equip them with the tools necessary for selecting and verifying the origin of information, its veracity and its quality. This could help users mitigate the problem of misinformation by helping them sort reliable and unreliable informational resources. But it could also support users in tackling the problem of persuasive design by making them aware of the biases which some informational resources exploit. Without information literacy seen as “a broad, integrated and critical perspective on the contemporary world of knowledge and information” (Shapiro and Shelley 1996), individuals would be unable to foresee and avoid the traps of persuasive design and dangers that come from taking at face value all informational resources on the Internet.

The issue with digital literacy is that it is often understood as an acquisition of technical skills for operating computers. Most of the time, digital literacy becomes an engineering class and is equated with the promotion “of more efficient uses of the medium – for example, via the development of advanced search skills (or so-called «power searching») that will make it easier to locate relevant resources amid the proliferation of online material” (Buckingham 2015, 24). To be successful, digital literacy must include a broader attempt of learning not only technical issues, but also how the new media and technologies help us represent the world, how the information we acquire through these technologies can be evaluated critically so as to produce knowledge. Besides the practical and analytical skills it bestows on individuals, information literacy should enhance users’ autonomy, by bridging the gap between them and various experts (from coders to information architects), offering the former at least a glimpse into how the digital world is created and works; which could be a first step in understanding not necessarily from a technical, but from a social and political point of view how information and power asymmetries affect each and every one of us. The purpose of digital literacy is to empower the individual in her use of the Internet, to acquaint her with the subtle ways in which the Internet is used and put to use for manipulation, disinformation or persuasion, and to give her the tools to avoid falling prey to these uses.

Despite its benefits, information literacy is not sufficient to prevent the detrimental effect of online information on individual cognitive abilities, due to the complexity of the computational environment we live in. The cognitive burden of selecting and acquiring reliable information could also be reduced by strengthening the collaboration between individuals for the purpose of filtering and ranking information. Our second strategy uses the “wisdom of the crowds” to bring some structure to online information.

Since the birth of Web 2.0, the participatory and collaborative nature of the online realm has enabled the emergence of new forms of collective intelligence. The notion of collective intelligence (CI) is based on the idea that intelligence emerges not only from individual human brains but also from groups of people. The most interesting forms of applied collective intelligence for our purposes are social tagging and crowdsourcing for assessing the reliability of online information. Social or collaborative tagging can be used for information management and organization; volunteers are encouraged to attach different representational terms (tags) to Internet-based resources which can be later shared with others. These classification systems thus rely on the aggregated efforts of individual users. Tags are basically labels which describe the content of a site or document, simplifying information searching and retrieval practices. Social tagging is extremely successful, like in the case of del.icio.us or Flickr (Smith 2008), and although the expectation is that the more people contribute to tagging, the more chaotic the resulting system will be, some studies have found the contrary (for example, Golder and Huberman 2006). The “wisdom of the crowds” is also very efficient in assessing the reliability of online information, thus helping tackle the problem of misinformation. A recent study showed that SNSs could counter the spread of misinformation by employing an aggregate assessment of websites’ reliability made by individual users (Pennycook and Rand 2019). This method proves more cost efficient and effective than employing professional fact-checkers. An example of successfully using crowdsourced taxonomies for assessing the reliability of informational sources is the browser add-on Web of Trust. This add-on warns users when they are accessing dangerous websites; these recommendations are the result of aggregating millions of users reviews and combining them with advanced ranking algorithms. Other examples are reputation systems based on individual ratings which are used by “sharing economy” businesses like Uber, or AirBnB, TripAdvisor, Imdb and so on. However successful these applications are in collecting and aggregating individuals’ opinions and evaluations, they are nonetheless domain specific; there are no “new levels of understanding” (Gruber 2008, 4) emerging. We can discover the best restaurants, hotels, drivers or movies, which points towards the fact that these systems of aggregation are generally applied in commerce, and tend to single out popularity which does not necessarily equate with quality.

Crowds are not infallible and they fail when people’s decisions are not independent from one another. In other words, when individuals are influenced by other’s opinions or guesses, there is a big chance that the aggregate effect will drift towards inaccuracy, which is precisely what happens in the case of eco chambers. This phenomenon is called the undermining effect of social influence (Lorenz et al. 2011) and its avoidance is crucial for the accuracy of the systems we propose. “Wisdom of the crowds” systems are particularly successful when the groups incorporate as many individual members as possible whose views are negatively correlated, meaning as different as possible from the views of the existing group members (Surowiecki 2005). Ensuring the diversity of opinions and worldviews by bringing together groups of politically, socially or religious individuals might be a successful way of tackling the detrimental effects of echo chambers. Accuracy is fostered by diversity of opinions because errors tend to cancel each other out.

The third strategy involves the creation of Artificial Intelligence (AI) companions meant to guide us to through the informational labyrinth. As it has been argued elsewhere (Savulescu and Maslen 2015, 80; Maes 1995), AI companions could be used to monitor and make us more aware of our actions online, and they could equally be put to the task of advising us about the dangers and threats encountered online. These digital assistants could help users with the problem of information overload, by helping them retrieve in a more efficient manner the information needed for a particular task. By employing machine learning techniques^7^ the artificial agent can gradually develop its abilities as it is trained by the user, while the user “is also given time to gradually build up a model of how the agent makes decisions, which is one of the prerequisites for a trust relationship” (Maes 1995). This means that the agent can retrieve the information the user teaches it to categorize as useful, which would considerably diminish the time spent searching for information. Such AI companions would thus work on rules developed alongside the user, unlike search engines which are based on pre-defined algorithms (see, for example, Introna and Nissembaum 2000). These AI companions could similarly cooperate with other similar entities on the network by sharing information about unreliable, manipulative or persuasive informational resources. The idea behind such automated personal assistants is that they could more easily detect security or privacy threats and they could warn users when spending too much time on a website which employs persuasive design.

The companions we propose could offer not only more effective ways of accessing information, but they could also function as guides that caution users of the dangers encountered, be they security, privacy or attentional threats. A benefit of using AI companions to navigate the Internet would be the potential to protect or even enhance users’ autonomy, which is threatened by the power and information asymmetries working in favor of the companies that provide the algorithmic governance of their digital life. But for this condition to be met, the automated personal assistants proposed must be open-source, for at least two reasons: firstly, open-source produces much better software than proprietary alternatives (Wheeler 2005), because anyone, anywhere can solve bugs or improve the software. Secondly, open-source gives control back to users, by providing all the necessary means to understand how the software works (thus eliminating the potential for surveillance, data collection, or breaches of privacy etc.) or even to control, customize or improve it. Open-source systems do not necessarily imply that users need to know or learn programming. The main advantage of making the AI open source is that users are theoretically free to understand it, to share, or even to modify it with the help of the others. All of these freedoms would be impossible under closed systems. Open systems are built around communities of programmers, testers, translators and other digital enthusiasts who offer their help to unexperienced users. As such, in order to benefit from an open-source system, it is not necessary that users themselves be technically savvy. There are many open-source systems (such as Ubuntu) which are user-friendly and intuitive, but because they are open, the potential for surveillance, data collection, or breaches of privacy is much smaller than in the case of closed systems.

## Conclusions

Although the Internet is one of the main drivers of change and evolution, its capacity to radically transform human cognition is exaggerated. No doubt this technology has improved numerous areas of our lives by facilitating access to and exchange of knowledge. However, its cognitive enhancement potential is not as clear as originally assumed. Too much information, misinformation, and the exploitation of users’ attention through persuasive design, could result in a serious decrease of users’ cognitive performance. The Internet is also an environment where users’ cognitive capacities are put under stress and their biases exploited.

Despite these warnings, our analysis is not committed to deep skepticism towards the value of the Internet as a whole. The focus was mainly on the social-semantic layer of the network, and on some of the issues generated by social networks and other information platforms. These are the most-used Internet based services with billions of active users. But the Internet can be put to many different uses in a number of different ways. Once we understand that the Internet is a malleable technology, we can imagine the creation of new communication protocols or layers which could avoid the problems of information processing, and secure users’ privacy and autonomy.^8^ If we want to further the debate about the enhancement potential of the Internet, we need to take a more integrative approach which highlights how technology is intertwined with our psychological and cognitive makeup. This is exactly the purpose of the three strategies proposed—improving information literacy, harnessing the “wisdom of the crowds” for information organization and categorization, and developing AI companions. Mere access to vast quantities of information is not enough to amount to robust cognitive enhancement. More is needed. Users must be empowered, their autonomy respected and their decision-making capacities enhanced for the technology to be considered a true enhancement.

## Footnotes

1 From a technical standpoint the Internet could be defined as a network of networks built upon a decentralized infrastructure composed out of an ever-growing number of interconnected nodes.

2 For a history of techno-optimism in approaching computers and the Internet see Winner (1984) and Wellman (2011).

3 For a history of the concept of information overload see Rosenberg (2003); for reviews on literature see Jacoby (1984), Eppler and Mengis (2004), Edmunds and Morris (2000), Hall and Walton (2004), Bawden et al. (1999). It was identified under different names such as cognitive overload (Kirsh 2000), sensory overload (Malhotra 1984), communication overload (Miller 1964), knowledge overload (Coates 2009) and information fatigue syndrome (Lincoln 2011).

4 The views equating cognition with computers are, strictly speaking, metaphors (Floridi 2011, 35–42). Computation and information processing are not the same, even though they are used interchangeably. Information processing is generally used to convey the methods in which organisms keep track of their environments, while computation refers to the process of acting on inputs under the control of a rule which is sensitive to some properties of the inputs, following some steps from one initial state to the output. The reason for the conflation of the two terms is most probably historical and has to do with the cybernetic school’s attempts at blending Shannon’s theory of information with Turing’s theory of computability (Piccinini and Scarantino 2011).

5 Hubert Dreyfus (2009, 13) was one of the first to draw the opposition between what he called the “old library culture” and the “hyperlinked culture”. For him, the former is based on classification, careful selection and permanent collections, while the latter is enormously diverse, it offers access to everything, and its collections are dynamic.

6 There is a distinction between misinformation and disinformation. While most researchers argue that false information is a contradiction in terms—information can only be true in some views, for example (Shannon 1948; Floridi 2011; Himma 2007)—they nonetheless make a distinction between data whose semantic content is false, which would be misinformation, and data whose semantic content is false and which is intentionally meant to deceive the receiver, meaning disinformation (Floridi 2011, 260). The difference between the two of them can be treated, then, in terms of intention and deception.

7 Especially supervised and reinforced learning, which can be based on previously labeled information gathered through collective intelligence mechanisms. This solution is feasible only if the AI development is open and thus scrutable.

8 Solid, a project to “save the World Wide Web”, is exactly such an attempt led by the inventor of the original web, Sir Tim Berners-Lee, at MIT. Its intention is to give back data ownership and control to users. Pretty similar in target is the employment of the blockchain technology to guard source authenticity, truthfulness and responsibility of information.
